# Quality of physical therapy from a patient’s perspective; factor analysis on web-based survey data revealed three dimensions on patient experiences with physical therapy

**DOI:** 10.1186/1472-6963-14-266

**Published:** 2014-06-18

**Authors:** Marijn Scholte, Hilly Calsbeek, Maria WG Nijhuis-van der Sanden, Jozé Braspenning

**Affiliations:** 1 Scientific Institute for Quality of Healthcare, Radboud university medical centre, Geert Grooteplein 21, 6525 EZ Nijmegen, The Netherlands

**Keywords:** Patient experiences, Factor analysis, Quality of care, Physical therapy

## Abstract

**Background:**

Assessing quality of care from the patient’s perspective has changed from patient satisfaction to the more general term patient experience, as satisfaction measures turned out to be less discriminative due to high scores. Literature describes four to ten dimensions of patient experience, tailored to specific conditions or types of care. Given the administrative burden on patients, less dimensions and items could increase feasibility. Ten dimensions of patient experiences with physical therapy (PT) were proposed in the Netherlands in a consensus-based process with patients, physical therapists, health insurers, and policy makers. The aim of this paper is to detect the number of dimensions from data of a field study using factor analysis at item level.

**Methods:**

A web-based survey yielded data of 2,221 patients from 52 PT practices on 41 items. Principal component factor analysis at item level was used to assess the proposed distinction between the ten dimensions.

**Results:**

Factor analysis revealed two dimensions: ‘personal interaction’ and ‘practice organisation’. The dimension ‘patient reported outcome’ was artificially established. The three dimensions ‘personal interaction’ (14 items) (median_practice level_ = 91.1; IQR = 2.4), ‘practice organisation’ (9 items) (median_practice level_ = 88.9; IQR = 6.0) and ‘outcome’ (3 items) (median_practice level_ = 80.6; IQR = 19.5) reduced the number of dimensions from ten to three and the number of items by more than a third.

**Conclusions:**

Factor analysis revealed three dimensions and achieved an item reduction of more than a third. It is a relevant step in the development process of a quality measurement tool to reduce respondent burden, increase clarity, and promote feasibility.

## Background

Quality of care from the patient’s perspective is increasingly in the spotlight, but what exactly does it mean? From the mid-80s onward, there has been a general shift in healthcare to view patients as consumers of care [1]. With that shift has come a notion that consumer satisfaction can serve to measure the quality of public health services [2,3]. Throughout the past decades, the same concept was studied, though with different names and with slightly different contents, from patient satisfaction, patient empowerment, patient-centeredness to patient experiences. Patient scores of satisfaction with certain aspects of healthcare proved hard to interpret, as the term satisfaction was not well defined and its simplicity did not acknowledge the multidimensional nature of satisfaction [4]. A shift was made from measuring the opinion of the patient to measuring facts to assess the quality of care. With that came a tendency to see the patient as a whole, autonomous person (patient-centeredness) who needed to be empowered to act as a full partner in the treatment process (patient empowerment) [5]. The more general term ‘patient experience’ arose around the same time and incorporated the former two terms. In this study, the latter term is used, as it does the most justice to the multidimensionality and complexity of quality of care from a patient’s perspective. Over time, there have been a lot of initiatives to measure patient experiences. An important survey for measuring quality of care from the patient’s perspective is the Consumer Assessment of Healthcare Providers and Systems (CAHPS), a programme of the U.S. Agency for Healthcare Research and Quality [6]. This survey captures patients’ experiences in four dimensions (receiving necessary care, receiving care quickly, how well doctors communicate, and customer service).

In the Netherlands, a national programme started in 2007 measuring the quality of physical therapy care. The programme was developed as consensus-based among patients, physical therapists, health insurance companies, as well as the Health Care Inspectorate [7]. Apart from the dimensions of the quality of a practice’s performance and that of the actual organisation, a tool was developed to assess the quality of care from a patient’s perspective. A modified RAND appropriateness Delphi procedure was used, in which evidence for the dimensions from a literature review were not sent to the experts, but rather the framework that was extracted from literature [7,8]. An agreement was reached in three rounds on ten quality dimensions from the patient’s perspective focusing on the following dimensions: accessibility, accommodation, information and communication, physical therapist’s approach, continuity, self-management support, intervention outcome, global perceived effect (GPE), length of intervention period, and patient-centeredness (see Table 1). A patient questionnaire covering 41 items was developed to measure these dimensions [7] (see Additional file 1).

**Table 1:** Proposed dimensions for patient experience: dimension, description and items measured

**No**	**Dimension**	**Description**	**Items measured**
1	Accessibility	The average degree (in%) of accessibility	access by phone; access by transport; free choice therapist; free choice appointment time; waiting time until first appointment; waiting time in practice (less than 15 minutes); appropriate treatment time; appropriate expertise (n = 8)
2	Accommodation	The average degree (in%) of accommodation requirements	hygiene; comfort (waiting and exercise room); enough chairs waiting room; privacy; accessibility (n = 6)
3	Information and communication	The average degree (in%) of perceived information and communication	open attitude to questions; clear explanations; tried to understand my problem; informed about course of disease; informed about intervention period; clear intervention; explained daily exercises; advised on daily life; fit between the actual and expected intervention period; results of treatment discussed (n = 10)
4	Physical therapist’s approach	The average degree (in%) of perceived physical therapist’s approach	empathy; politeness; attentive listening; taken seriously; feeling at ease; taking into account specific needs (n = 6)
5	Continuity	The average degree (in%) in continuity	treatment by more than one therapist; adequate preparation; consistency of information; progress discussed with general practitioner (n = 4)
6	Self-management support	The average degree (in%) in perceived self- management support	working together to reach intervention goals; advice to prevent new complaints; monitoring the accuracy of the exercises at home; monitoring the adherence to the advice given (n = 4)
7	Intervention outcome	The average degree (in%) in which the intervention outcome is reached	increased performance in daily activities; fit between actual and expected intervention outcome (n = 2)
8	Global perceived effect (GPE)	The average degree (in%) in which the outcome in terms of the GPE is reached	*Global Perceived Effect* scale (GPE) (n = 1)
9	Length of intervention period	The average degree (in%) in which the length of the intervention period is as expected	fit between actual and expected intervention period (n = 1)
10	Patient-centeredness	The average degree (in%) of patient- centeredness	free choice therapist (see 1); appropriate expertise (see 1); privacy (see 2), GPE score (see 8), fit between actual and expected outcome (see 7); discussed different treatment methods

In these patient surveys, high item scores combined with low variance [3] raised questions about the usability of patient experiences to measure differences in quality and of using the patient’s perspective as an instrument to improve the quality of care. In other words, does the knowledge gained equal the weight of the burden that is placed on the patients? In the meta-analysis by Hush, Cameron, and Mackay [3] for example, the average satisfaction rate of patients in physical therapy was 4.44 out of a five-point scale with a 95% confidence interval of 4.41–4.46. With such high scores and low variance, it becomes very difficult to distinguish high performing practices from practices with lower quality of care. As a consequence, these measurements are not appropriate for pay-for-performance strategies of insurance companies or as consumer information to guide choices between health care providers.

Low variance has been associated with the length of the questionnaire [9], as respondents become bored and fatigued with long surveys and less willing to put effort into answering questions. More uniform answers are given in longer surveys, affecting the variance in the data. Related to this is the lack of consensus regarding the definition of separate dimensions, and thus on the number of items needed. Four to ten dimensions are described in literature that should capture patient experiences with health care [10-13]. The reduction of the number of dimensions, resulting in a decrease of the number of items, and thus a lessening of the burden placed on the respondents, should be part of the development process to ensure the collection of high quality data.

In this study, whether the consensus-based dimensions that measure patient experiences of physical therapy in primary care can be statistically identified will be tested along with whether or not item reduction is possible. The dimensions of quality measurements are often evaluated through an examination of their internal consistency; a factor analysis at item-level to clarify the number of dimensions is much less common. Testing the internal consistency of the dimensions separately will not show whether the distinction between dimensions was justified to begin with. Factor analysis at item level will show if the same dimensions can be extracted from the data. The aim of this study therefore is to perform an exploratory factor analysis at item-level to detect the number of dimensions in patients’ experiences with physical therapy.

## Methods

### Study population and data collection

A group of primary care physical therapy practices (n = 52) volunteered to participate in a field test in 2008. Physical therapists (n = 292) were asked to invite 40 of the most recent patients who had finished their treatment by means of a standardised letter with a unique log in code for the web portal to complete the questionnaire on patient experience (n = 2,221 patients). The physical therapists also received a poster and folder material for the patients regarding the project. In the survey the respondents were instructed to tick the box of the appropriate answer. If the question was not applicable to them, or if the respondent did not know the answer, they were instructed to tick the box ‘not applicable’ or ‘I don’t know’. The study was conducted in accordance with the Declaration of Helsinki. The CMO (Committee on Research Involving Human Subjects) of the Arhem and Nijmegen region decided that the study did not fall within the scope of the WMO (Act Medical Research on Human Subjects) and could be performed without assessment of the CMO, since the questionnaires were not onerous or extensive to such an extent that they would substantially interfere with the daily lives of the participants.

The data for calculating the dimensions were retrieved using a web-based system with a portal, electronic questionnaires, and a feedback function. After data collection, each practice received a feedback report with the dimension scores of the practice and the individual therapists as well as the median scores of all participating practices as a benchmark.

### Statistical analyses

The dimension scores were calculated as the ratio of the sum of the scores of the rated items to the total of possible items scores [8] (See Additional file 1 for an overview of the questions and answer categories). All items were recoded, resulting in a high item score corresponding to a high level of quality^a^. The calculation was performed at patient level and then transformed to physical therapist level and practice level by determining the patient’s median score per therapist and practice. A dimension score was only calculated if the respondent had valid scores on all items of that particular dimension. Descriptive statistical analysis was used to summarise the dimension scores at practice level. Principal Component Analysis was applied with promax rotation (oblique) to all unique *items*, to test how many dimensions in patient experience could be established. Only factor loadings of 0.4 and higher were considered relevant, and to properly interpret them, items should only load 0.4 or higher on one component. Further, an Eigen value of > 1 was used to consider a component. All statistical tests were performed in SPSS version 20, and for all statistical tests a significance level of p < 0.05 was used.

## Results

### Study population

The population of the field study was a representative sample of Dutch patients visiting a physical therapist with respect to gender, direct access vs. referred patients, and acute vs. chronic patients (see Table 2). However, elderly patients (65 years and older) were underrepresented, as were patients aged 24 years and younger [14].

**Table 2:** Patient characteristics in comparison to representative sample

		**Patient experience (n = 2,213 patients)**	**National representative sample 2010**^**12 **^**n = 13,180 patients**
		**%**	**%**
Gender patients	male	37.7	40.4
Age patients	0-11 years	1.7	2.7 (0–14 y)
	12-18 years	4.4	
	19-24 years	4.5	18.8 (15–24 y)
	25-44 years	32.1	26.5
	45-64 years	44.8	37.7
	65 years and older	12.6	22.2
Direct access		32.2	42.6
Chronic (more than 18 sessions)		18.8	16.7

### Dimension scores

Overall, median scores at *practice level* were high (see Table 3) with relatively low variation. Dimension 2 (‘accommodation’) and 4 (‘physical therapist’s approach’) scored highest with a median score at practice level of 100. Dimension 5 (‘continuity’) and dimension 8 (‘global perceived effect’) showed the most room for improvement of quality.

**Table 3:** Dimension scores at practice level: N, median, minimum score, maximum score and Interquartile range (IQR)*

**Dimension**	**N**	**Median**	**Min**	**Max**	**IQR**
1: Accessibility	50	91.7	83	96	4.7
2: Accommodation	51	100	72	100	5.6
3: Information and communication	52	90	77	97	6.3
4: Physical therapist’s approach	52	100	89	100	0
5: Continuity	52	66.7	42	100	8.3
6: Self-management support	52	83.3	63	92	8.3
7: Intervention outcome	47	88.3	50	100	16.7
8: Global perceived effect (GPE)	47	75	50	100	25
9: Length of intervention period	47	83.3	33	100	33.3
10: Patient-centeredness	51	85.4	74	89	6.3

IQR = third quartile – first quartile.

### Dimensions

Some of the items were used in more than one dimension as it was discussed that a single dimension should be valid in itself, and thus an item could ‘complete’ more than one dimension. However, in factor analysis an item can only be contributed to a single component. The items in the analysis are therefore unique items (n = 41).

The items monitoring the results of the treatment (3 items: items no. 39, 40, 41 Additional file 1) were analysed separately, due to their distinct difference in meaning from the other items. Factor analysis on these three items showed high loadings on a single component (Table 4) and the newly constructed dimension ‘outcome’ created a Cronbach’s alpha of 0.73, which is acceptable.

**Table 4:** Obliquely rotated component loadings for 3 items on outcome

**Dimension**	**Item description**	**Component 1**
7	Improvement in performing daily activities	0.761
7	Fit between result and expected result	0.842
8	Global perceived effect (GPE)	0.825

In the factor analysis of the remainder of the unique items (n = 38), 13 components were extracted with an Eigenvalue > 1, explaining almost 60% of the total variance (see Table 5). There were 10 items that loaded <0.4, and since a clear indication as to which component they belonged to was not possible for these items, they were discarded and excluded from Table 5. Items 1 through 14 all loaded high on component 1. All items were linked to the concept ‘personal interaction’, of which the scale produced a Cronbach’s alpha of 0.81. The items mainly came from the presupposed dimensions ‘communication and information’ ‘physical therapist’s approach’ and ‘self-management support’. Although items 15 through 17 also loaded high on component 1, they also loaded above 0.4 on component 3, and are therefore less clear to interpret. For that reason, they will be discarded for the construction of the above-mentioned dimension ‘personal interaction’. Items 18 through 26 all loaded on different components and mainly came from the presupposed dimensions ‘accessibility’ and ‘accommodation’. They did, however, have conceptual coherence, meaning they were all related to the concept of ‘practice organisation’. However, the items did not correlate, which explains why they did not load on the same component. A change in the item ‘access of practice by phone’ led to a change in score of the dimension, but not the other way around. A change in score on the dimension ‘practice organisation’ did not lead to a change in score of each item, which would be the case if the items were effect indicators [15]. The group of items therefore can be considered as causal indicators of the latent concept or dimension [15] ‘practice organisation’, on which no reliability of scale test can be performed. Further, items 27 and 28 loaded high on other components than component 1 as well, although they lacked a comparable conceptual meaning. They will therefore not be included in the dimension ‘practice organisation’. One option is to treat them as separate dimensions, but this would give them too much weight. For that reason, the items will be discarded.

**Table 5:** Obliquely rotated component loadings for 37 items*

**Dimension**	**Item no. and description**	**Component**												
		**1**	**2**	**3**	**4**	**5**	**6**	**7**	**8**	**9**	**10**	**11**	**12**	**13**
1	1. Appropriate treatment time	0.52												
3, 7 & 10	2. Fit between the actual and expected intervention period	0.48												
3	3. Tried to understand my problem	0.44												
3	4. Informed about course of disease	0.63												
3	5. Explained daily exercises	0.60												
3	6. Advised on daily life	0.64												
3	7. Open attitude to questions	0.40												
3	8. Clear explanations	0.43												
3	9. Clear intervention	0.53												
4	10. Empathy	0.43												
4	11. Attentive listening	0.57												
4	12. Taken seriously	0.47												
4	13. Taking into account specific needs	0.60												
4	14. Working together to reach intervention goals	0.58												
4	**15. Feeling at ease**	**0.50**		**−0.43**										
6	**16. Monitoring the accuracy of the exercises at home**	**0.55**		**0.51**										
6	**17. Monitoring the adherence to the advice given**	**0.60**		**0.50**										
1	18. Access by phone									0.43				
1 & 10	19. Free choice therapist								0.40					
1	20. Waiting time in practice												0.66	
1 & 10	21. Appropriate expertise				0.44									
1	22. Accessibility practice					0.42								
2	23. Comfort exercise room		0.51											
2	24. Comfort waiting room										−0.64			
2	25. Comfortable chairs in waiting room					0.57								
5	26. Treatment by more than one therapist											0.49		
3	27. Informed about intervention period		−0.44											
5	28. Consistency of information				0.41									

*Only loadings >0.4; **Bold** items are discarded as they load above 0.4 on more than one component.

To summarise, three dimensions could be statistically distilled; ‘personal interaction’ (14 items) (median_practice level_ = 91.1; IQR = 2.4), ‘practice organisation’ (9 items) (median_practice level_ = 88.9; IQR = 6.0) and ‘outcome’ (3 items) (median_practice level_ = 80.6; IQR = 19.5). The new dimensions were calculated in the same manner as the ten proposed dimensions, namely as the ratio of the sum of the scores of the rated items to the total of possible items scores.

## Discussion

The aim of this study was to test how many dimensions in patient experiences with physical therapy in primary care could be distilled. Factor analysis showed that the ten proposed dimensions within patient experience can be reduced to three, and as a result the number of items can be reduced by 15, which is more than a third.

The reduction of dimensions from ten, sometimes overlapping dimensions to three clear and easy to interpret dimensions creates clarity for health care professionals, who can now see at a glance in what areas they can improve their services, as well as for patients for whom the information on the quality of care is easier to comprehend. Last, the item reduction makes the survey more feasible, putting less of a burden on the patients. Further research is needed to assess the quality of the shorter version of the questionnaire.

The dimensions found are comparable to the results of other studies in the field. Concurrent with the field test in this study, the CAHPS was introduced to the Netherlands, and so-called Consumer Quality Indices (CQI) were developed for several conditions and care settings [16]. The CQI uses three dimensions to measure patient experiences with health care providers (‘conduct of health care providers’, ‘access to care’, ‘receiving the care needed’). The dimension ‘conduct of health care providers’ is comparable to the dimension ‘personal interaction’ in this study, although the CQI only uses five items [13] of which four are exactly the same as those found in this study. The key area ‘relationship with the professional’ distinguished by May in his review of patient satisfaction in management of back pain [10] and the dimension ‘clinical behaviour’ (of general practitioners) found by Wensing [17] are also comparable to ‘personal interaction’. Wensing [17] uses 16 items (including two on outcome) of which half are comparable to our items, and the other half are occupation specific for general practitioners. Further, the dimension ‘interpersonal care’ (of general practitioners) found by Bower, Mead and Roland [18] covers eight items, of which five are practically the same as in the current study. May’s review [10] further distinguished a key area ‘environmental issues’, which can be compared to the dimension ‘practice organisation’ in the current study covering access and facilities components. Wensing [17] found ‘organisation of care’ (seven items), Bower, Mead and Roland [18] found the dimension ‘access’ (five items) and De Boer et al. [13] found ‘access to care’ (eight items). Again, about half of the items of these studies are similar to the items for ‘practice organisation’ in the current study. May’s [10] was the only study to distinguish a separate key area on ‘clinical outcome’. Wensing [17] incorporated outcome in the dimension ‘clinical behaviour’, whereas the others did not mention outcome at all.

The concepts of personal interaction and organisational aspects are largely agreed upon in literature, with some differences in content as well as in the number of items needed to form the scale. The aim should always be to minimise strain on the patients while maintaining quality information. Further research on item reduction in quality dimensions of patient experience is needed to achieve this goal. The main difference within literature is of the dimension ‘outcome’, which was treated as a separate dimension in the present study. As May [4] points out, a positive outcome is not always correlated to a satisfied patient and should therefore be measured separately. Further, patients who seek the best treatment for their conditions might value information on outcome scores of health care providers.

One of the major limitations regards data collection. Selection bias might have played a role in this study, as physical therapists themselves recruited the patients for participation . It was clear that the information from this survey could have financial consequences for physical therapy practices in the future, since health insurance companies are making a shift from paying for quantity to paying for performance. It is therefore conceivable that physical therapists selected patients suffering from less complex problems, for example, who were treated successfully, or with whom they had good communicative relations. There are roughly three other ways to collect data from patients. The first option is to have a permanent collection. However, the high scores on the dimensions of patient experiences do not justify such a time-consuming effort, both for patients and physical therapists. A second option is to randomly select patients for invitations from the databases of health insurance companies or directly from the Electronic Medical Records (EMRs), for example. A third way is to compare the experiences of patients with measurements of the quality of the physical therapy process of the same patients. Measuring the quality of physical therapy care from a patient’s perspective was part of a broader attempt to monitor the quality of physical therapy care as a whole. Besides patient experiences, the quality of the clinical reasoning process with respect to the screening and diagnostics process, the intervention process, and the outcome, was also measured [7]. This survey was based on the existing guidelines concerning the necessary steps in the clinical reasoning process and was completed by the physical therapists. If this data could be extracted directly and randomly from the EMRs, and if the selected patient cases could also be invited to participate in the patient experience survey, the results could be compared. Assessing the same process from different perspectives can be very valuable, since understanding differences in perceptions between therapist and patient can help the professional to better understand the needs of the patients they are treating and thus improve the (perceived) quality of care. However, as this has never been described in literature to the knowledge of the authors, more research is necessary to establish the added value to measuring and ultimately improving the quality of care.

Secondly, most quality dimensions are developed through a consensus-based process. Consensus is a very important first step to create a basis for quality research and the development of quality measurements. Involving all stakeholders can create the support base necessary to ensure participation of all parties involved. Besides this, a good starting point is to prioritise subjects with a broad scope and to discuss what is important for patients. In this way, ten dimensions of patient experiences were proposed to be tested in the field. Statistical testing should be part of the development process. It is often seen however, as was the case in our study, that quality programmes have already been nationally introduced while, in the meantime, information is still collected on the testing properties. Pressure from stakeholders to supply data is high. Still, this study has shown that factor analysis is a valuable next step in the development process as it can redefine and sharpen the proposed dimensions of quality of care from a patient’s perspective. In trying to satisfy the patients and to meet their needs, the consensus procedure has led to an overestimation of the number of dimensions patients distinguish, as the analysis showed, even though patient organisations were involved in the development process. Sharpening the definitions of the dimensions of the patient’s perspective will help to better measure the quality of care. Further, it becomes clearer where possibilities for the improvement of the quality of care lie. Finally, patients do not benefit from too many, vaguely formulated dimensions, but with three clear dimensions they can compare practices with ease on the dimensions they value the most.

Lastly, only a small number of patients who participated in the data sampling had finished their treatments (n = 350), although this was a requirement in the instruction to the physiotherapists. This means that most of the patients were still being treated, a situation that could also lead to bias, as the patients still depended on the physical therapists. It also means that the items measuring outcome were calculated on a small proportion of patients instead of the patient sample as a whole. This last limitation could be a result of the relatively short period of data collection.

A compelling question, given the high scores and low variance, is whether or not patients should be bothered with surveys on the quality of care at all, as the CQI, for instance, produced very high scores and low variance as well [13]. Further studies need to examine whether the reduced length of the questionnaire increases variance and thus increases the quality of the data. However, there are other ways to monitor quality of care, or to extract the bad apples. The quality of ‘personal interaction’ can also be monitored by having a mandatory open-access complaint registration. However, studies of such complaint systems within hospitalised care conclude that a lot of adverse events are unreported by patients and health professionals [19]. Therefore, a combination with other forms of quality measurement is necessary, such as a combination with a shorter survey on patient experiences every three years or so to ensure sufficient information on the quality of care, thereby minimising the strain on patients. Practices can be audited at all times by the Inspectorate, should the complaint registration or low performance scores on the patient experience survey give rise to concerns on the quality of care. ’, It is questionable whether the patient should be asked to evaluate the dimension ‘practice organisation’ as well. To assess the most basic organisational requirements, certifications can serve as quality measurements just as well as asking patients, if not better. Since a lot of the physical therapy practices already have a certification, why ask the patients as well? One problem with this is that the certifications cost a lot of money and time. Besides this, they are not mandatory, so practices can choose not to participate.

Based on the above, we recommend a thrice-yearly, shorter survey of triangulated patients who are randomly selected from the EMRs. Besides this, a visible and mandatory complaint desk (physical or digital) should be implemented to monitor the quality of care at all times. If need be, the Inspectorate can audit the low performing practices based on the number of complaints or low performance on the surveys.

## Conclusions

Three dimensions of patient experiences with physical therapy in the Netherlands were extracted from the data of the field study, i.e. ‘personal interaction’, ‘practice organisation’ and ‘outcome’, reducing the number of proposed dimensions from ten to three and the number of items needed by more than a third. This study shows that factor analysis is a relevant step in the development process, as the reduction of dimensions and items will increase clarity for health care professionals and patients and it will promote feasibility. Future research should focus on testing the shortened questionnaire and trying to triangulate quality data, both from the health professional’s perspective and the patient’s perspective. Ultimately, transparency in the quality of care is best served by high quality information that is easy to interpret for all stakeholders.

## Endnote

^a^The item Global Perceived Effect (item 41 Additional file 1) was recoded so that categories 5–9 were rated the lowest quality and category 1 the highest quality.

## Competing interests

The authors declare that they have no competing interests.

## Authors’ contributions

MS participated in the design of the study, performed the statistical analysis, and drafted the manuscript. HC helped to draft the manuscript. MWGN and JB conceived of the study, participated in its design and coordination, and helped to draft the manuscript. All authors read and approved the final manuscript.

## Pre-publication history

The pre-publication history for this paper can be accessed here:

http://www.biomedcentral.com/1472-6963/14/266/prepub

## Supplementary Material

Additional file 1 **All items used in factor analysis with full questions and answer categories.** Description: All items used in factor analysis with full questions and answer categories. Click here for file
