# Supplementary material for: Quality of physical therapy from a patient’s perspective; factor analysis on web-based survey data revealed three dimensions on patient experiences with physical therapy
Source: BMC Health Serv Res. 2014 Jun 18;14:266. doi: 10.1186/1472-6963-14-266 (PMC4074141; doi:10.1186/1472-6963-14-266)
Supplement: Additional file 1 — All items used in factor analysis with full questions and answer categories. Description: All items used in factor analysis with full questions and answer categories. [file 1472-6963-14-266-S1.pdf]

Additional file 1. All items used in factor analysis with full questions and answer categories

Item no.	Question	Answer categories
1	How often did your physiotherapist spent enough time on you in treatment?	{1,Never}<br>{2,Sometimes}<br>{3,Usually}<br>{4,Always}
2	Did (does) the duration of the treatment (so far) correspond to your expectation?	{1,Not}<br>{2,Slightly}<br>{3,Largely}<br>{4,Fully}
3	To what extent did your physiotherapist try to understand the problem for which you came?	{1,Not}<br>{2,Slightly}<br>{3,Largely}<br>{4,Fully}
4	Were you well informed by your physiotherapist about the probable course of your complaint or illness?	{1,Never}<br>{2,Sometimes}<br>{3,Usually}<br>{4,Always}
5	How often did your physiotherapist clearly explained how, why, and how often an exercise had to be carried out at home?	{1,Never}<br>{2,Sometimes}<br>{3,Usually}<br>{4,Always}<br>{5,I didn't have home exercises}*
6	How often did your physiotherapist give you advice that you could daily use?	{1,Never}<br>{2,Sometimes}<br>{3,Usually}<br>{4,Always}
7	How often was your physiotherapist available during treatment for questions?	{1,Never}<br>{2,Sometimes}<br>{3,Usually}<br>{4,Always}
8	How often did your physiotherapist explain things in an understandable way?	{1,Never}<br>{2,Sometimes}<br>{3,Usually}<br>{4,Always}
9	How often did your physiotherapist clearly explain what he/she was doing?	{1,Never}<br>{2,Sometimes}<br>{3,Usually}<br>{4,Always}
10	Did your physiotherapist regularly ask how you were doing?	{1,Never}<br>{2,Sometimes}<br>{3,Usually}<br>{4,Always}
11	How often did your physiotherapist listen to you carefully?	{1,Never}<br>{2,Sometimes}<br>{3,Usually}<br>{4,Always}
12	How often did your physiotherapist take you seriously?	{1,Never}<br>{2,Sometimes}<br>{3,Usually}<br>{4,Always}
13	How often did your physiotherapist take your specific circumstances and wishes into account?	{1,Never}<br>{2,Sometimes}<br>{3,Usually}<br>{4,Always}
14	Did your physiotherapist work together with you to achieve the treatment goals?	{1,Not}<br>{2,Slightly}<br>{3,Largely}<br>{4,Fully}

*Coded system missing

Additional file 1. Continued

15	How often did your physiotherapist ensure that you were feeling at ease with him/her?	{1,Never}<br>{2,Sometimes}<br>{3,Usually}<br>{4,Always}
16	Did your physiotherapist ask you if your exercises at home went well?	{1,Never}<br>{2,Sometimes}<br>{3,Usually}<br>{4,Always}<br>{5,I didn’t have home exercises}*
17	Did your physiotherapist ask what you had done with his/her advise?	{1,Never}<br>{2,Sometimes}<br>{3,Usually}<br>{4,Always}<br>{5,I did not receive advise}*
18	How often was the physiotherapy practice accessible by phone?	{1,Never}<br>{2,Sometimes}<br>{3,Usually}<br>{4,Always}<br>{5,I have not called the practice}*
19	Did you have the possibility to choose a physiotherapist that suited you?	{1,No}<br>{2,Yes}
20	How often were you helped within 15 minutes of the time of the appointment?	{1,Never}<br>{2,Sometimes}<br>{3,Usually}<br>{4,Always}
21	Was there sufficient specialist expertise for your needs available within the physiotherapy practice?	{1,Yes}<br>{2,No}<br>{3,I had no need for specialist treatment}*<br>{4,I don't know}*
22	Was the physiotherapy practice easily accessible with the transportation that you usually used?	{1,Never}<br>{2,Sometimes}<br>{3,Usually}<br>{4,Always}
23	Did you think that the exercise room offered sufficient comfort (with regard to size, temperature, light, floor surface)	{1,Never}<br>{2,Sometimes}<br>{3,Usually}<br>{4,Always}<br>{5,Does not apply}*
24	Did the practice have facilities to make the wait more pleasant for you?	{1,Yes}<br>{2,No}<br>{3,Does not apply}*
25	Did you think that there were enough comfortable seats present in the waiting area of the practice?	{1,Never}<br>{2,Sometimes}<br>{3,Usually}<br>{4,Always}
26	Have you been treated by different physiotherapists for your complaints?	{1,No}<br>{2,Yes}
27	Were you well informed by your physiotherapist about the duration of the treatment?	{1,No}<br>{2,Yes}
28	How often did your physiotherapist give you contradictory information?	{1,Never}<br>{2,Sometimes}<br>{3,Usually}<br>{4,Always}

*Coded system missing

Additional file 1. Continued

29	Within how many days after registration could you go to your physiotherapist?	{1,Within 0-2 days}<br>{2,Within 3-7 days}<br>{3,Within 8-14 days}<br>{4,Longer than 14 days}
30	How often could you make an appointment at a time that suited you?	{1,Never}<br>{2,Sometimes}<br>{3,Usually}<br>{4,Always}
31	Did you think that the physiotherapy practice was clean?	{1,Never}<br>{2,Sometimes}<br>{3,Usually}<br>{4,Always}
32	Did you think that the practice offered enough privacy? (For example, when changing clothes, during consult, during the treatment and regarding confidentiality of data)	{1,Yes}<br>{2,No}
33	Was the physiotherapy practice accessible for you? (For example with regard to the entry of the practice, were there (too) high thresholds, or difficult steps)	{1,No}<br>{2,Yes}
34	Did the physiotherapists inform you on different treatment possibilities?	{1,No}<br>{2,Yes}<br>{3,Does not apply to my complaints}*
35	How often did your physiotherapist treat you polite?	{1,Never}<br>{2,Sometimes}<br>{3,Usually}<br>{4,Always}
36	Did your physiotherapist discussed the progress of your treatment with your general practitioner (in consultation with you)?	{1,No}<br>{2,Yes}<br>{3,I don't know}*<br>{4,Does not apply}*<br>{5,I have indicated myself that I would not appreciate feedback to my general practitioner }*
37	Did your physiotherapist give you advise on how you could prevent new complaints?	{1,No}<br>{2,Yes}
38	Were the results of the treatment discussed with you?	{1,No}<br>{2,Yes}
39	How often did you feel that you could perform your daily activities better because of the treatment of your physiotherapist?	{1,Never}<br>{2,Sometimes}<br>{3,Usually}<br>{4,Always}
40	To what extent did (does) the result of the treatment (up to now) correspond to your expectations?	{1,Not}<br>{2,Slightly}<br>{3,Largely}<br>{4,Fully}
41	To what extent did your complaints change in relation to the period before the treatment? The complaints are ...	{1,Extremely better}<br>{2,Much better}<br>{3,Moderately better}<br>{4,Slightly better}<br>{5,No difference}<br>{6,Slightly worse}<br>{7,Moderately worse}<br>{8,Much worse}<br>{9,Extremely worse}

*Coded system missing
